# Series AC Arc Fault Detection Method Based on High-Frequency Coupling Sensor and Convolution Neural Network

**DOI:** 10.3390/s20174910

**Published:** 2020-08-31

**Authors:** Ruobo Chu, Patrick Schweitzer, Rencheng Zhang

**Affiliations:** 1 Key Laboratory of Process Monitoring and System Optimization for Mechanical and Electrical Equipment (Huaqiao University), Fujian Province University, Xiamen 361021, China; cruobo@stu.hqu.edu.cn; 2 Institut Jean Lamour (IJL), CNRS, University of Lorraine, F-54000 Nancy, France; patrick.schweitzer@univ-lorraine.fr

**Keywords:** series arc, fault diagnosis, high-frequency coupling sensor, convolution neural network

## Abstract

Arc faults induced by residential low-voltage distribution network lines are still one of the main causes of residential fires. When a series arc fault occurs on the line, the value of the fault current in the circuit is limited by the load. Traditional circuit protection devices cannot detect series arcs and generate a trip signal to implement protection. This paper proposes a novel high-frequency coupling sensor for extracting the features of low-voltage series arc faults. This sensor is used to collect the high-frequency feature signals of various loads in series arc state and normal working state. The signal will be transformed into two-dimensional feature gray images according to the temporal-domain sequence. A neural network with a three-layer structure based on convolution neural network is designed, trained and tested using the various typical loads’ arc states and normal states data sets composed of these images. This detection method can simultaneously accurately identify series arc, as well as the load type. Seven different domestic appliances were selected for experimental verification, including a desktop computer, vacuum cleaner, induction cooker, fluorescent lamp, dimmer, heater and electric drill. Then, the stability and universality of the detection algorithm is also verified by using electronic load with adjustable power factor and peak factor. The experimental results show that the designed sensor has the advantages of simple structure and wide frequency response range. The detection algorithm comparison confirms that the classification accuracy of the seven domestic appliances’ work states in the fourteen categories could reach 98.36%. The adjustable load in the two categories could reach above 99%. The feasibility of hardware implementation based on FPGA of this method is also evaluated.

## 1. Introduction

Arcing can be considered as a complex electromagnetic reaction process. According to the Under Laboratories (UL) Standard UL1699 [1], an arc fault is defined as a continuous luminous discharge of electricity across an insulating medium and usually accompanied by the partial volatilization of the electrodes. It is caused by the breakdown of the insulation medium in the electrical circuit, the loosening of the electrical connection, the humidity of the air, the reduction of the insulation strength. The high temperature generated by the arc fault discharge phenomenon is very easy to ignite the surrounding combustibles, which is one of the main causes of electrical fires.

According to the statistics of the national fire and rescue situation of the Fire and Rescue Bureau of the Chinese Ministry of Emergency Management in 2019 [2], a total of 233,000 fires were reported throughout the year, causing direct property losses of 463 million dollars. Although urban and rural residential fires accounted for only 44.8% of the total, the number of casualties caused by the year accounted for 78.3% of the total. In residential fires, electrical fires are still high. In total, 52% of the fires that have been identified are caused by electrical causes, especially the fires caused by various household appliances and electrical lines.

In July 2013, the International Electrotechnical Commission (IEC) published the international standard IEC 62606:2013 [3]. In 2014, the American Electrical Code (NEC) [4] further required installing a combined AFCI in all 15A and 20A circuits in residence. By then, China promulgated the national standard GB/T 31143-2014 [5] and GB14287.4-2014 [6].

The arc fault detection device (AFDD) is a new type of circuit protection device to detect and identify the arc fault in an electrical network, and make a trip or alarm action in time to prevent electrical fires caused by the arc. An arc fault is an unintentional dangerous arc in electrical circuitry. According to their location in the circuit, arc faults are categorized into three types: ground arc fault, parallel arc fault, and series arc fault.

Parallel arc fault and ground arc fault are usually caused by short circuits between power lines, which have higher current magnitude. So the traditional short circuit or over current protection systems can detect and make protective actions more effectively. In contrast, the current magnitude of series arc fault is limited by the load in the circuits. Series arc fault’s current is generally the same as the normal working current, or even slightly smaller. As a result, the traditional short-circuit, over-current, overload, and leakage protection technologies cannot effectively detect the series arc fault in the circuit. Therefore, the emergence of AFDD and arc fault detection technology makes up for the vacancy in the closed-loop protection system of electrical safety.

When an arc fault occurs in power distribution network, many physical quantities that can characterize it such as arc sound, arc light, arc voltage, and arc temperature [7,8]. However, because the location where the arc fault occurs is unknown, the detection of the above physical parameters is more difficult in practical applications because the sensors used to measure these physical parameters must be installed near the position where the fault arc occurs. The convenience of current measurement makes it an ideal parameter for arc fault detection.

Over recent years, relevant researchers have conducted extensive research on series arc fault detection.

The research on the mathematical model of arc fault is helpful to understand the generation principle of arc fault, and it is also the basis of arc fault identification. Liu et al. [9] and Qu et al. [10] improved the Mayr and Cassie arc models and optimized the parameters of the original arc models. The simulation results are closer to the experimental results.

The assumption of arc model research is stable arcing or high current. Due to the innovation of various household appliances, especially the use of switching appliances widely, the arc fault in low-voltage distribution systems becomes more complex, which makes the research on the model of low-voltage arc faults mostly stay in the simulation stage.

The interests of arc fault diagnosis applications based on machine learning such as discrete wavelet analysis (DWA), support vector machines (SVM), and artificial neural network (ANN) is increasing [11,12,13,14]. In [15], the source data are sampled at a rate of 25 kHz for analyzing five typical loads. According to the linear harmonic combination of the loads, these loads were divided into three classes. By then, a specific fully connected neural network is designed for each class to identify the arc. Yang et al. [16] analyze the frequency distribution of arc fault features when series arc faults occurred in different loads, and pointed out that the frequency distribution characteristics of arc fault mainly is concentrated in the frequency band of 2 kHz to 20 MHz. In [17,18], the voltage waveform at the power source is used to implement series arc fault detection. Tisserand et al. [19] propose a method for identifying series arcs in an AC or DC electrical installations by the algebraic derivative of the current. Lezama et al. [20] present an embedded system for series arc detection by evaluating the inter-period correlation coefficient of the line current to determine if an arc fault has occurred.

These methods [11,12,13,15,16,17,18,19,20] determine a trigger threshold according to the change of extracted eigenvalues. They are effective in some applications, but the diversity of load makes them lack of reliability and generalization ability.

Most of the literature reported that the frequency band of arc feature signal extraction is within 200 kHz [15,20]. Arc current is an ideal parameter for arc fault detection but the measurement frequency band of common current transformer (CT) and hall sensor (HS) is generally from 0 to 200 kHz [21,22]. Another reason is that the processing ability of the embedded system is generally less than 1 MHz. However, it must be noted that the frequency components above 200 kHz are still abundant.

Hence, a novel high-frequency coupling (HFC) sensor is designed for extracting the features of low-voltage series arc faults. It has the advantages of simple structure and wide frequency response range A series arc fault acquisition experimental platform was built, and the HFC sensor is employed to collect the high-frequency feature signals of various loads in arc state and normal state. The signal will be transformed into a two-dimensional gray image. A three-layer artificial neural network based on convolution neural networks is designed to accurately identify series arc and load, as well as the load type.

The following parts of the paper is divided into five sections. Section 2 presents the characteristics of HFC sensor including working principle, equivalent circuit, frequency response, etc. In Section 3, the features on series current in time and frequency domain for typical appliances under different working states would be analyzed. Section 4 designs a detection methodology named HCCNN and introduces its framework, working procedure, and the method for generating data using the HFC sensor. Section 5 designs the test bench and evaluates the performance of the proposed method with experiments on data sets. Section 6 presents a comparison between the HCCNN and prior methods. Section 7 is devoted to the conclusion.

## 2. HFC Sensor

In this section, the structure and principle of HFC sensor are introduced, and the transfer function of sensor is analyzed and discussed.

### 2.1. Equivalent Circuit and Principle Analysis of the HFC Sensor

**Figure 1:**
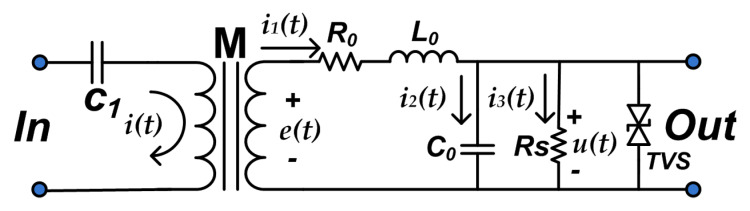
High-frequency coupling sensor schematic.

The schematics of the HFC sensor is shown in Figure 1. It consists of a polypropylene film capacitor $C_{1}$, a common-mode inductance *M*, a sampling resistor Rs, and a pair of transient voltage suppression diodes TVS. $R_{0}$ and $L_{0}$ are the internal resistance and self-inductance of the sensor respectively, while $C_{0}$ is its parasitic capacitance. The common mode inductor *M* is used as the isolation unit between strong and weak signals. The left coil of *M* and $C_{1}$ constitute a high pass filter, by which the fundamental frequency 50 Hz of power supply will be filtered out. The TVS ensures that the following circuit components and high-speed acquisition equipment are protected from the transient high-energy impact caused by arc fault.

The value of the mutual inductance of the *M* (which represents the magnetic coupling). Depends on the geometry, size, relative position, number of turns of its two coils, and the magnetic permeability of the medium around. The mutual inductance can be measured by experiment. The induced electromotive force generated by mutual inductance can be expressed by (1).

(1) $$e\left(t\right)=M\frac{di(t)}{dt}$$

According to Kirchhoff voltage and current theorem, the expression of (2) and (3) can be derived from Figure 1:

(2) $$e\left(t\right)=R_{0}i_{1}\left(t\right)+L_{0}\frac{di_{1}\left(t\right)}{dt}+u\left(t\right)$$

(3) $$i_{1}\left(t\right)=i_{2}\left(t\right)+i_{3}\left(t\right)=C_{0}\frac{du(t)}{dt}+\frac{u(t)}{R_{s}}$$

Since the ferrite core used for the coupling have high value of inductance, which is far greater than the impedance of internal resistance $R_{0}$ and sampling resistance Rs. The current flowing through $C_{0}$ is much smaller than that flowing through Rs, because the value of $C_{0}$ is small. In summary, Equations (4) and (5) can be obtained.

(4) $$L_{0}\frac{di_{1}\left(t\right)}{dt}\gg R_{0}i_{1}\left(t\right)+u\left(t\right)$$

(5) $$\frac{u(t)}{R_{s}}\gg C_{0}\frac{du(t)}{dt}$$

The simplification of (2)–(5) gives (6). It can be noted that the output voltage of the HFC sensor has a linear relationship with the input current of the common mode inductor. The input side of the sensor is a high pass filter, so the measured current is an alternating current.

(6) $$u\left(t\right)\approx \frac{MR_{s}}{L_{0}}i\left(t\right)$$

### 2.2. Frequency Response Analysis of HFC Sensor

**Figure 2:**
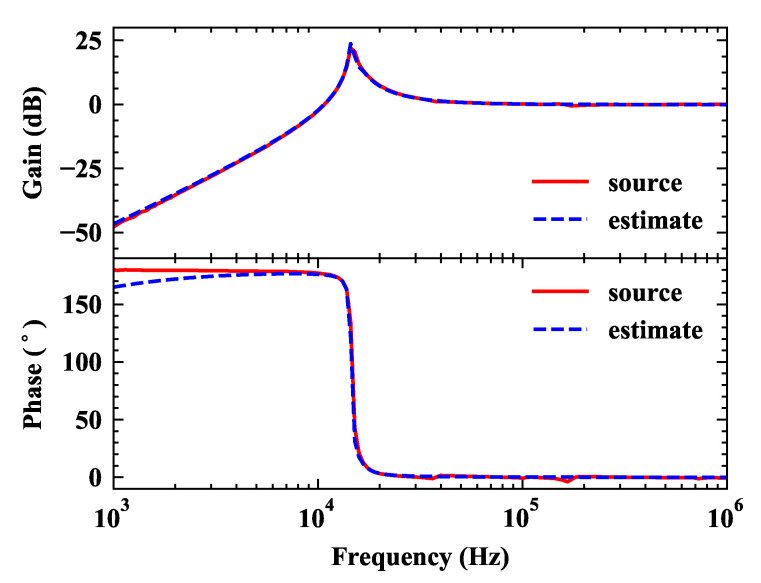
The frequency response analysis (Bode) of the HFC sensor.

The most important parameters of HFC sensor are the bandwidth and sensitivity, which both can be obtained from the frequency response curve. The frequency response analysis chart was measured by oscilloscope (DSOX-1204G, Keysight Technologies, Santa Rosa, CA, USA). According to the source data measured by oscilloscope, the transfer function is estimated. The frequency response curve and its estimate curve are shown in Figure 2. The sensor contains a two-order oscillation section and its corner frequency is around 13 kHz. Equation (7) shows the general form of the estimated transfer function of HFC sensor.

(7) $$H\left(s\right)=\frac{a_{0}s^{3}+a_{1}s^{2}+a_{2}s+a_{3}}{b_{0}s^{3}+b_{1}s^{2}+b_{2}s+b_{3}}$$

where $a_{0}$, $a_{1}$, $a_{2}$, and $a_{3}$ are −1.01, $7.76\times 10^{2}$, $-2.08\times 10^{9}$, and $1.09\times 10^{13}$, respectively. $b_{0}$, $b_{1}$, $b_{2}$, and $b_{3}$ are 1.00, $-8.45\times 10^{4}$, $8.17\times 10^{9}$,and $-7.64\times 10^{14}$, respectively. A high pass filter can be found by analyzing the frequency response of the HFC sensor whose low-frequency cut off frequency is around 10 kHz. When the frequency is 1 kHz, its frequency response is close to −50 dB.

The HFC sensor is designed to connected to a low-pass filter in series to form a specific band-pass filter sensor. Details of the design are discussed in the next section

## 3. Time and Frequency Characteristic Study

In this section, seven different domestic appliances were selected for investigation, including desktop computer, vacuum cleaner, induction cooker, fluorescent lamp, dimmer, heater, and electric drill. Their waveforms in time domain and frequency domain and their grayscale feature image generated from HFC sensor will be analyzed to get multiple perspectives for further identification.

### 3.1. Time Domain

**Figure 3:**
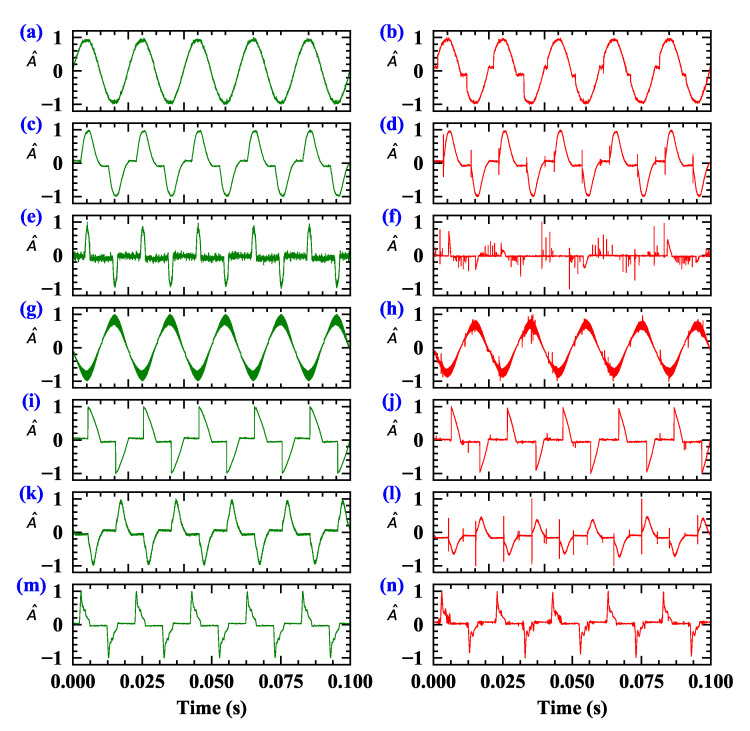
The current waveforms of seven representative appliances in normal and arcing state. Sub-graphs in the left column (green curve) are the current waveform in normal state, while the right (green curve) column are that of arcing state. (**a**,**b**) Heater, (**c**,**d**) Vacuum cleaner, (**e**,**f**) Desktop computer, (**g**,**h**) Induction cooker, (**i**,**j**) Dimmer, (**k**,**l**) Electric drill, (**m**,**n**) Fluorescent lamp.

Figure 3 shows the current waveforms, which have been normalized, for the two circuit states (normal and arcing) from different typical appliances. Current signatures can be downloaded freely on Zenodo. Subgraphs in the left column is the current waveform in normal state, while the right column is that of arcing state. The loads’ working current is obviously different due to different load types. Figure 3a is the normal working current of the electric heater, with complete sine waveform. When an arc fault occurs, abnormal behaviors such as “flat shoulder” in the zero-crossing, increment of the harmonic component and amplitude distortion impulse might be observed. Particularly, the normal state of dimmer load that has very pronounced “flat shoulder” could imitate the arc state of electric heater, causing misjudgment. An electrical drill may produce several arc features at zero crossing, the same as the vacuum cleaner. The randomness of current variation can also be mimicked by the desktop computer load. Whether in the arc state or normal state, the load of induction cooker has rich frequency components.

Therefore, methods only based on the analysis of the appliances’ current generally cannot content with the requirements of accurate and reliable of arc fault detection, on account of the variety and complexity of arcing.

### 3.2. Frequency Domain

**Figure 4:**
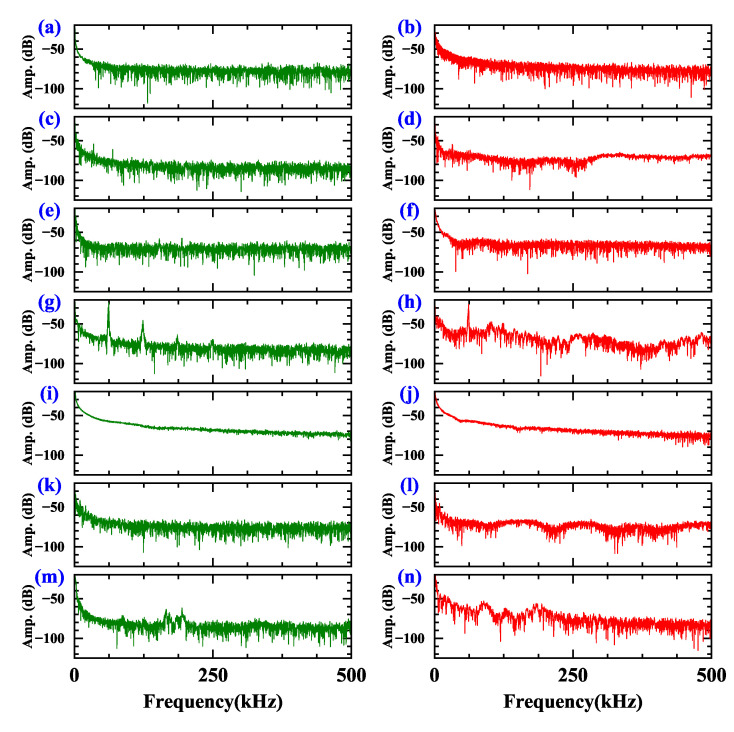
The frequency spectrum distribution of current in normal and arcing state. Sub-graphs in the left column (green curve) are the current waveform in normal state, while the right (green curve) column are that of arcing state. (**a**,**b**) Heater, (**c**,**d**) Vacuum cleaner, (**e**,**f**) Desktop computer, (**g**,**h**) Induction cooker, (**i**,**j**) Dimmer, (**k**,**l**) Electric drill, (**m**,**n**) Fluorescent lamp.

In order to eliminate the bias issue of instruments and eliminate the influence of power between different load types, the sampling current is Max-Min normalized by (8) before frequency domain conversion.

(8) $$y_{i}=\frac{x_{i}-\min_{1\leq j\leq N}\left\{x_{j}\right\}}{\max_{1\leq j\leq N}\left\{x_{j}\right\}-\min_{1\leq j\leq N}\left\{x_{j}\right\}}$$

where $\left\{x_{j}\right\}$ represents the current time series input, *N* is the number of elements in a current series, and $\left\{y_{i}\right\}$ represents the result. The expression of fast Fourier transform (FFT) in discrete form is illustrated by (9).

(9) $$z_{k}=|\sum_{m=1}^{N}y_{m}e^{-i\frac{2\pi }{N}km}|;\;1\leq k\leq N$$

where $\left\{y_{m}\right\}$ represents the normalized current time series input,$\left\{z_{k}\right\}$ is the result of FFT operation, and *N* is the number of elements in a current series. The current is sampled at a rate of 1 MHz with a digitizer. Figure 4 gives the spectrum distribution of seven domestic appliances in the arcing and normal current.

It should be noted that the features of arc appear in both low-frequency and high frequency region. In arcing state, the low frequency component for the majority of appliances decreases, while the high-frequency component increases. The change of heater that belongs to the resistive load is the high-frequency region mainly between normal and arcing state. The dimmer is off more than half of the cycle, and its power is relatively small, resulting in a thin line after transform. In arcing state, the frequency of inductive load (vacuum cleaner and electric drill) and induction cooker load shows irregular wave shape.

The spectrum of diverse load types is different in normal and arcing state. If there is switching load or dimmer load in the circuitry, even in normal state, its current also contains quite a lot of harmonic components. It is unreliable to diagnose the arc according to the change of frequency component value in a specific frequency band.

### 3.3. Gray-Scale Image Generated from HFC Sensor

Seven domestic appliances are sampled with the designed HFC sensor, and the sampling rate is 1 MHz. If the frequency of the residential power distribution network is 50 Hz, 10,000 sampling data will be obtained from every half-cycle, which is considered an identification object. The signal collected by the HFC sensor will generate the feature image through the following three steps. Firstly, the signal data (half-cycle, 10,000 data) are converted into absolute values and normalized. Then the data are mapped to the two-dimensional gray space of 0–255, as shown in (10):

(10) $$s\left(i,j\right)=255-255\frac{x_{100(j-1)+i}-\min_{1\leq n\leq N}\left\{x_{n}\right\}}{\max_{1\leq n\leq N}\left\{x_{n}\right\}-\min_{1\leq n\leq N}\left\{x_{n}\right\}}$$

**Figure 5:**
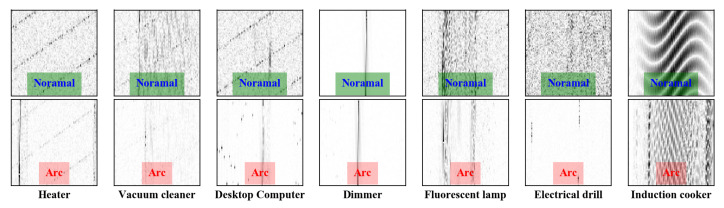
Gray image of of seven representative loads in normal and arcing state generated from HFC sensor. The normal state sub-graphs are in the first row, and the arcing state sub-graphs are in the second row.

Finally, the gray image is transposed for better observation and analysis. This step is not necessary and will be removed from the hardware implementation. As shown in Figure 5, each column represents a kind of load. The normal state sub-images are in the first row, and the arc state sub-images are in the second row.

These sub-images have their own features. For example, there are two stripes on the left and right sides of the arc state image of the heater, which is caused by the “flat shoulder” features of the heater in the time domain, while heater image in the normal state has obvious harmonic fringes and high-frequency noise. The feature image of the electric drill in the arc state will appear pinstripes randomly. However, in the normal state, it is full of noise. The feature images of fluorescent lamps have wide stripes, but there are some harmonics and bottom noise in normal images. The computer characteristic image also has this kind of appearance, but the stripe is lighter. When the induction cooker is in the arc state, its regular image will be destroyed by the arc. With respect to the dimmer, there are obvious cut-off fringes in the middle of the characteristic image of the dimmer due to the switch action of the silicon tube.

**Figure 6:**
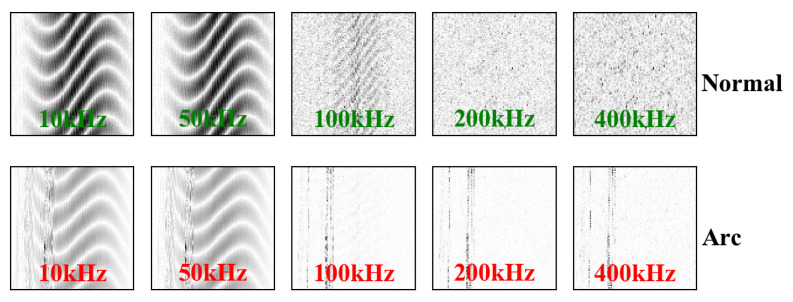
Gray images of induction cooker in normal and arcing state after filtering by a high pass filter with different cut-off frequencies.

**Figure 7:**
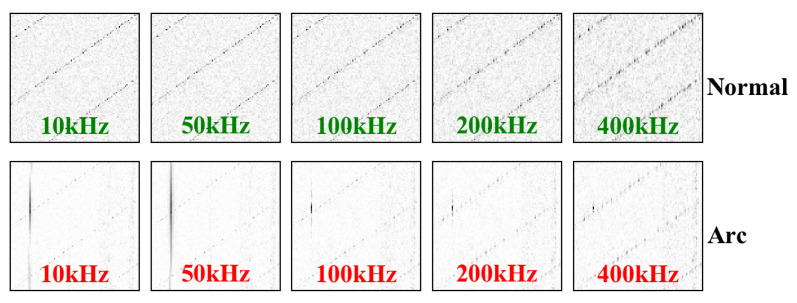
Gray images of heater in normal and arcing state after filtering by a high pass filter with different cut-off frequencies.

Figure 6 and Figure 7 show the grayscale images of the electrical heater and induction cooker passing through a high pass filter with different cut-off frequencies in normal and arcing state. With the increase of the cut-off frequency of the high pass filter, the background signal brought by the load itself is eliminated in the arc gray image.

If the cut-off frequency is 400 kHz, the characteristic high-frequency signal of the arc becomes weaker, but still exists. Therefore, we can draw a conclusion that although the spectrum of characteristic signal generated by arc fault is vast wide, the rich characteristic information is mainly concentrated within 400 kHz.

## 4. The HCCNN Method

In the previous section, through the analysis of the gray image generated by the HFC sensor, it is found that the images of various loads have their characteristics, which enlightens the development of the arc recognition algorithm. The high-frequency coupling convolutional neural network (HCCNN) method is proposed for series arc fault detection. In this section, the structure of the HCCNN method and its parameters will be introduced in detail.

### 4.1. Preliminary Theory of CNN

The deep convolution neural network (CNN) has the characteristics of feature extraction and data dimension reduction [23]. The basic structure of CNN includes input layer, convolution layer, pooling layer, full connection layer, and output layer. The convolution layer uses multiple moving convolution kernels to learn high-dimensional features from the image and performs the convolutional operation in a certain stride along with the input signal in horizontal and vertical directions. For an image x(i,j) with one channel, the convolution operation $F_{conv2D}\left(\right)$ is as follows:

(11) $$F_{conv2D}=\sum_{m}\sum_{n}x\left(i+m,j+n\right)w\left(m,n\right)$$

(12) $$y_{i}^{\left(l\right)}=f_{activ.}\left(b_{i}^{\left(l\right)}+F_{conv2D}\left(W_{i}^{\left(l\right)},y_{i}^{\left(l-1\right)}\right)\right)$$

where $y_{j}^{\left(l\right)}$ is the result of convolution, *W* is the weight coefficient matrix, *B* is the bias coefficient, and $f_{activ.}\left(\right)$ is the activation function. The pooling layer (lower sampling layer) [23] is usually placed after the convolution layer, which can not only retain the feature information extracted from the previous layer but also significantly reduce the amount of calculation. The output of the convolutional layer is the input of fully connected layer. The essence of the fully connected layer is matrix multiplication, which can be expressed as follows:

(13) $$y_{i}=f_{activ.}\left(W^{T}x_{i}+b\right)$$

where $f_{activ.}\left(\right)$ is the activation function. For multi-classification problems, the softmax activation function, as shown in (14), is generally employed. $\left\{x_{i}\right\}$ represents the input from the upper layer, $\left\{y_{i}\right\}$ is the prediction probability, and *W* and *B* represent the weight coefficient and bias coefficient, respectively.

(14) $$f_{softmax,i}\left(x_{i}\right)=\frac{e_{i}^{x}}{\sum_{j=1}^{N}e_{j}^{x}}$$

### 4.2. The HCCNN’s Structure

**Figure 8:**
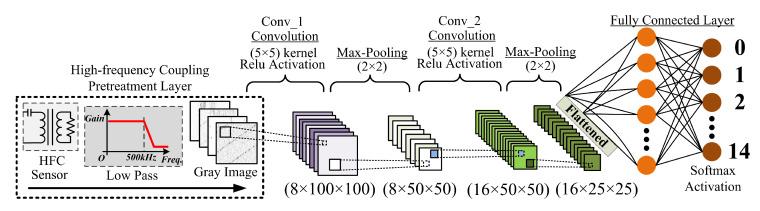
Composition of the HCCNN method.

The HCCNN method consists of a high-frequency coupling pretreatment layer, a convolutional network (ConvNet) layer, and a fully connected layer, as shown in Figure 8. These three layers work together to distinguish the type of load and diagnose from arc fault that occurs in the circuitry. The detailed parameters and architectures of the CNN layer and fully connected layer are summarized in Table 1.

The high-frequency coupling pretreatment layer consists of a HFC sensor and a low-pass filter, both of which are combined to collect signals in the frequency band (10 kHz–500 kHz) in the circuit. Then, these feature signals are processed into gray images. The convolutional network layer has two sub-convolution layers with 16 and 32 convolution kernels, respectively. This layer also uses the max pooling operation and the Relu activation function. The expression of the Relu activation function is as follows:

(15) $$f_{ReLU}\left(x\right)=\left\{\begin{array}{cc} 0, & x\leq 0,\\ 1, & x>0. \end{array}\right.$$

The artificial neural network (ANN) optimization procedure is minimizing the loss function as close to 0 as possible by training with an amount of data. For the HCCNN method, the cross entropy loss function is employed and its expression [24] is as follows:

(16) $$Loss=-\frac{1}{N}\sum_{n=1}^{N}\sum_{i=1}^{M}y_{n,i}\mathrm{lg}P_{n,i}$$

where $P_{n,i}$ is the output of the fully connected layer, *N* is the size of the input batch, $y_{n,i}$ is the corresponding targets, and *M* is the number of categories. The parameters are updated iteratively by the gradient of the loss function to the parameters. The gradient *g* requires computing:

(17) $$g\leftarrow \frac{1}{N}{▽}_{W,b}\sum_{n=1}^{N}Loss\left(f_{activ.}\left(x^{\left(n\right)};W,b\right),t^{\left(n\right)}\right)$$

where Loss is the per-batch loss function, *N* is the size of the mini batch, ▿ is the derivative operation, *W* and *b* are the weight and bias matrices, respectively. The gradient *g* and learning rate(lr) work together to update the parameters of neural network. This paper uses the Adam optimizer [25] to optimize the gradient update process.

(18) $$W^{*},b^{*}\leftarrow W,b-lr\cdot g$$

## 5. Experimental Setup and Results

In this section, experiments are carried out to evaluate the performance of the proposed HCCN method. A database consisting of seven domestic appliances was built for training and testing the HCCNN method. In order not to lose generality, the database generated by the electronic load is constructed.

### 5.1. Experimental Setup

**Figure 9:**
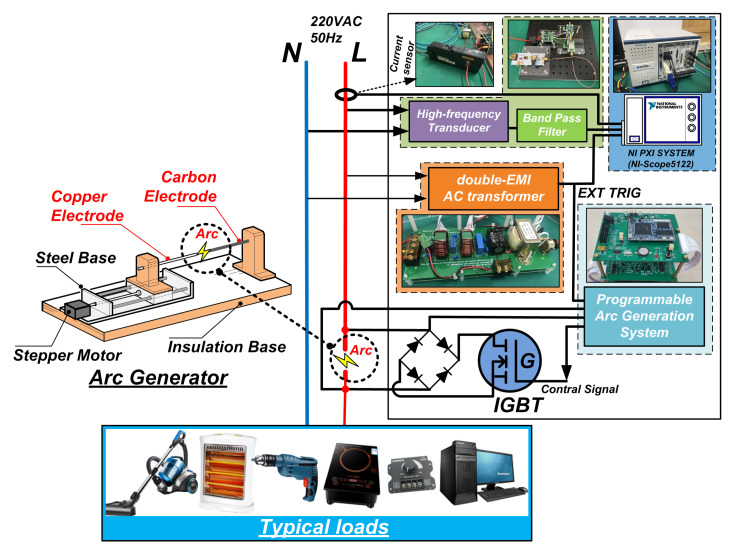
Schematic of the experimental platform.

In order to construct a large number of labeled normal samples and fault samples for the convolution neural network, a series arc fault acquisition experimental platform was built according to the Chinese standard GB14287.4-2014 [6]. The schematic of experimental platform is shown in Figure 9 The main equipment includes an adjustable arc generator with carbon and copper as electrodes, HFC sensor, PXI Chassis (PXIe-1071, National Instruments, Austin, TX, USA), the digitizer module (PXI-5122, National Instruments, Austin, TX, USA), AC power supply (IT-7626, ITECH, Nanjing, China) and current probe (N2783B, Keysight Technologies, Santa Rosa, CA, USA). A double series EMI filter is used for providing the external trigger signals for the digitizer to collect half-wave data completely, because of the frequency fluctuation of power grid.

**Figure 10:**
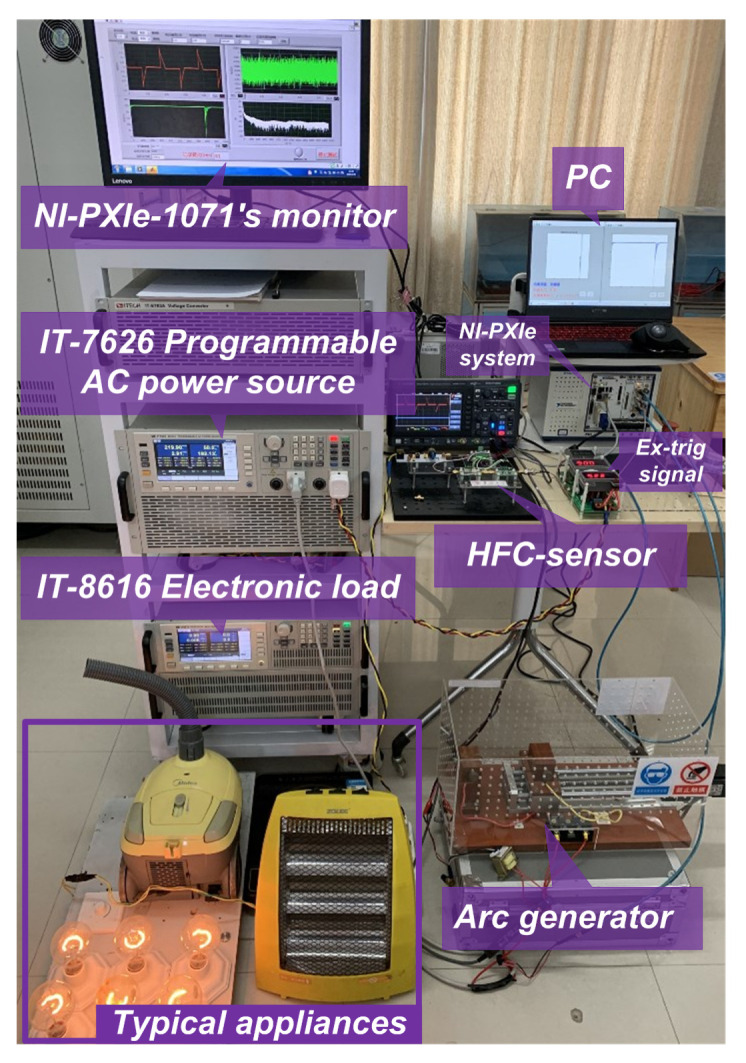
The test bench.

The arc generator is ignited between a carbon electrode and a copper electrode, as shown in Figure 9, is designed. A stepper motor with a programmable logic controller is used to accurately control the distance between electrodes with suitable speed. An IGBT device is connected to two electrodes of arc generator to precisely control the number of series arc by programmable arc generation system. Figure 10 represents the actual test bench.

The computers used for data processing is a computer with Intel CORE i7-9750H CPU and 16 GB DDR4 memory. The training and test procedure are primarily implemented within PyTorch and achieve comparable results.

### 5.2. The Arc Fault Database of Typical Appliances

Seven different domestic appliances were selected for experimental verification, including desktop computer, vacuum cleaner, induction cooker, fluorescent lamp, dimmer, heater, and electric drill. Seven typical appliances, corresponding powers, and manufacturers for this platform are illustrated in Table 2. All raw data are digitized at a sampling rate of 1 MHz using the HFC sensor and each half-cycle signal is considered to be a complete identification object. Because the frequency of the residential power distribution network is 50 Hz, each half-cycle signal will contain 10,000 sampling data, which will be converted into a gray image with a resolution of 100 × 100 pixels. The fully connected layer of the HCCNN method has fourteen output neurons, which correspond to fourteen categories from seven typical appliances.

Each load can be in arc state or normal state. In each state, 1000 half-wave data are collected to form 1000 gray images. Because there are seven typical domestic appliances for experimental verification, the database has a total size of 14,000 gray images. In total, 10% of the images to build the test set were randomly selected, so the size of the test set is 1400, while the size of the training set is 12,600.

### 5.3. Analysis HCCNN’s Output with t-SNE Method

**Figure 11:**
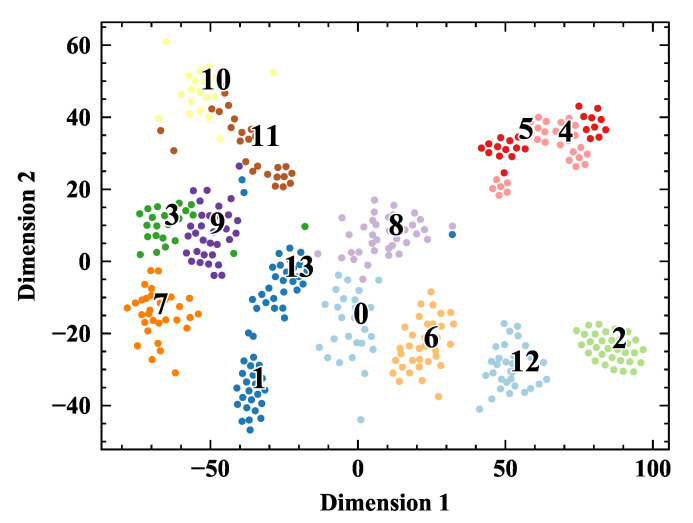
Visualization of the ConvNet layer’s output using the t-SNE method.

t-SNE method [26] is employed to analyze the output of the HCCNN’s ConvNet layer. The output of ConvNet layer is the high-dimensional features of image after convolution operation. The t-SNE method can map each sample into two-dimensional space, and try to separate each category from each other as far as possible. Figure 11 gives the dimension reduction distribution of the output of the ConvNet layer for seven domestic appliances. The load corresponding to the label of color point group can be found in Table 2. Odd labels are in arc state (1, 3, 5, 7, 9, 11, 13), and even labels are in normal state (0, 2, 4, 6, 8, 10, 12). The 14 load states shrink into a group and are far away from each other, and even tags and odd tags tend to separate from each other, which indicates that ConvNet layer has a good ability to extract load characteristics and arc features. However, some points are calculated to the wrong location, indicating that a fully connected layer is required for deeper classification.

### 5.4. The Detection Result of Typical Appliances

**Figure 12:**
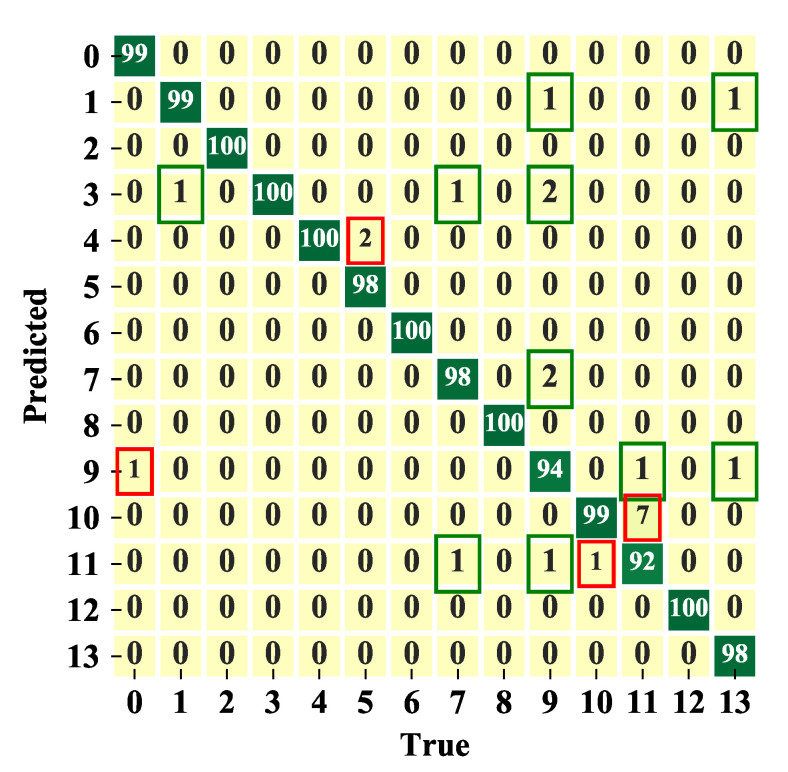
Confusion matrix of the test set.

The general distribution of the classification confusion matrix of the test data set with exact values is shown in Figure 12, in which labels and corresponding loads can be found in Table 2. The green borders indicate that the prediction of the working state is correct, but the prediction of the type is wrong. The red borders indicate that both the working state and the type of appliance are mispredicted.

Table 3 gives the classification results of the HCCNN method. The general prediction accuracy of each sample is 98.36%. In particular, the samples in green borders can be regarded as correct prediction if only concern weather arc fault occurs and ignore the type of appliance. If the type of appliance is not considered, the prediction accuracy of arc detection is further improved to 99.20%.

### 5.5. The Result of Electronic Load Recognition

Without loss of generality, the HCCNN method is evaluated using a test data set generated by an electronic load (IT-8616, ITECH, Nanjing, China) with adjustable power factor (PF) and CF (CF refers to the ratio of the peak value to the effective value of the periodic waveform). The CF is set to 1.45, 2.0, 2.5, 3.0, 3.5, 4.0, 4.5 and 5.0. The PF is set to 0.3, 0.4, 0.5, 0.6, 0.7, 0.8, 0.9 and 1.0. The overall size of the test set is 1600, of which 800 samples are arc state and the other 800 samples are normal state. Under the condition of each parameter, 50 normal and arcing state samples were collected. A script for recording and counting labels will be used to convert fourteen neurons in the HCCNN’s output layer into the arc or normal state.

**Figure 13:**
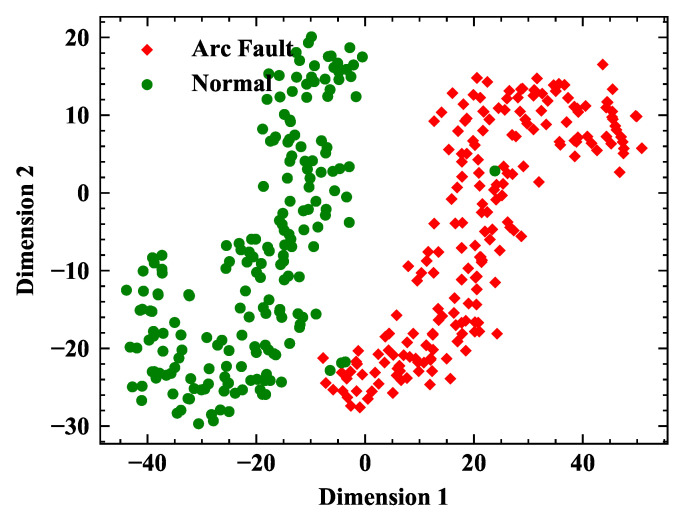
Visualization of the ConvNet layer’s output using t-SNE method.

Figure 13 gives the t-SNE distribution by testing database composed of feature images from the electronic load. The distribution that the selected 500 samples of normal and arcing state cluster into different areas reflects the extensive ability of HCCNN method. The overall recognition accuracy of the test samples is more than 99%.

**Figure 14:**
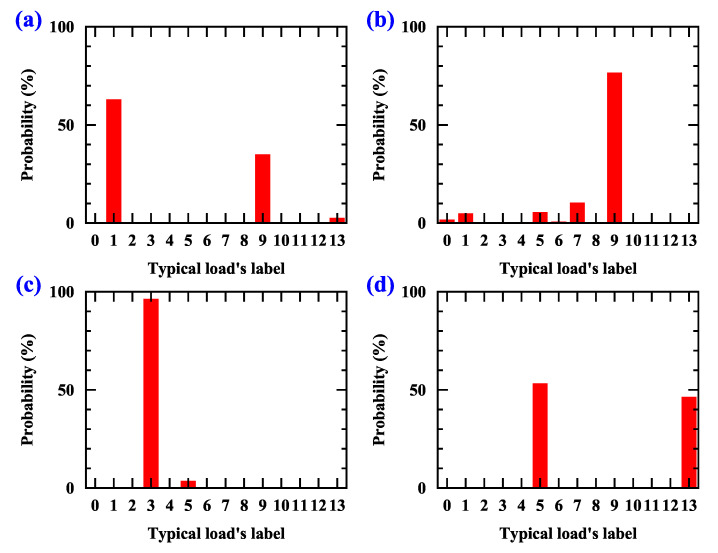
The probability distribution of the combination of two typical loads. (**a**) Heater and fluorescent lamp, (**b**) Dimmer and fluorescent lamp, (**c**) Vacuum cleaner and desktop computer, (**d**) Electric drill and desktop computer.

However, in the multi load’s parallel experiment, the HCCNN can accurately identify the arc and the load type as the load which accounts for the larger proportion of current in the circuit, but the accuracy will be reduced. The probability distribution of the combination of two typical loads is shown in Figure 14. Four experiments of two load combination (heater and fluorescent lamp, dimmer and fluorescent lamp, vacuum cleaner and desktop computer, electric drill and desktop computer) are used to verify the recognition results of the HCCNN method. For example, the heater load and fluorescent lamp load are combined and an arc fault occurs in the circuit. The predicted value of the heater is about 60% and that of the fluorescent lamp is about 30%. With a high power, the heater can be considered as a masking load.

### 5.6. Assessment of Embedded System Implementation

**Figure 15:**
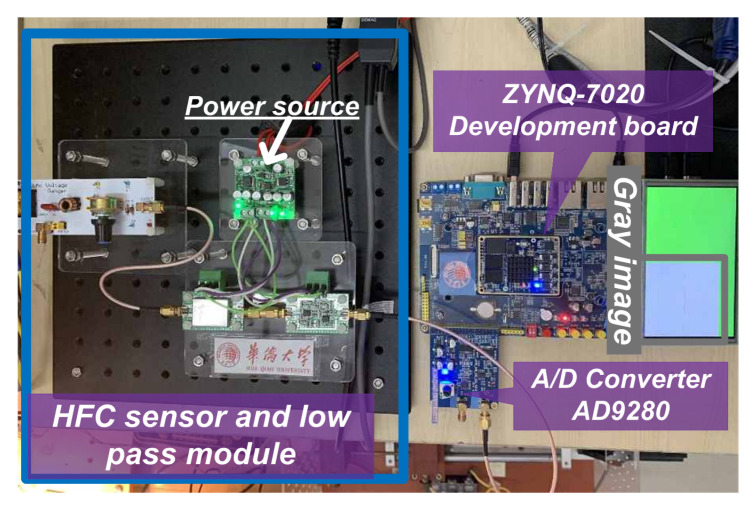
Embedded system test bench.

The implementation of the HCCNN method is based on a development board NAVIGATOR with a Zynq-7020 chip (xc7z020clg200, Xilinx, San Jose, CA, USA), which is a System-on-Chip (SoC) with a dual-core ARM Cortex-A9 processor and FPGA. Embedded system also employs a high-speed AD (AD9280, Analog Devices, Norwood, MA, USA), which is an 8-bit, 32 MSPS (sampling million times per second) analog-to-digital converter. The embedded system test bench is shown in the Figure 15.

**Figure 16:**
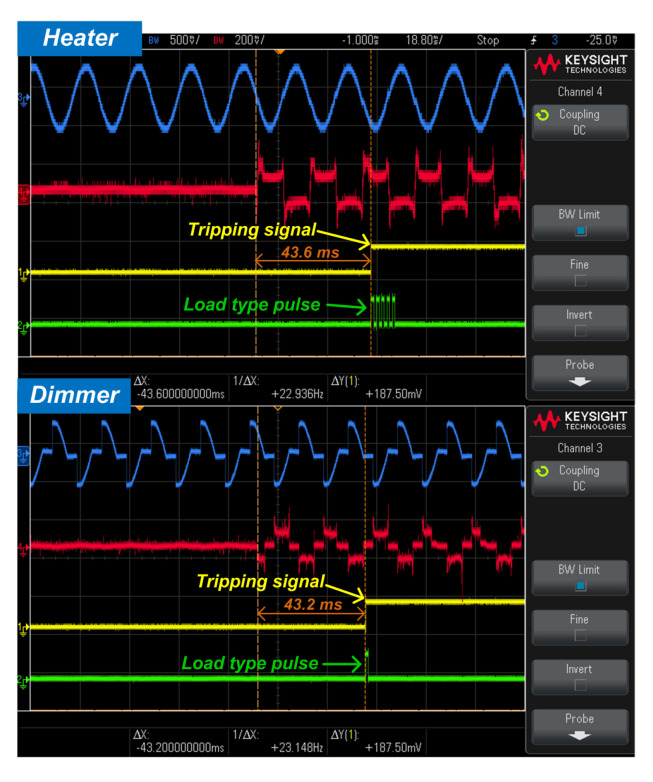
Examples of real-time experimental validation results using dimmer load and electrical heater: loop current (blue), voltage at both sides of the arc (red), tripping signal (yellow), load type pulse (green).

Figure 16 shows an experimental real-time result. The HCCNN method is able to accurately detect series arc fault generated by the electrical heater and dimmer. The embedded arc detection system will not only send out the tripping signal of the series arc fault but also send out the pulse of the corresponding load type. The proposed diagnosis scheme can detect series arc events at around 40 ms. In fact, a decision is done only after collecting a complete cycle signal, so the average detection time of every half cycle is about 10ms, meeting the requirements of detection time and having a significant margin.

## 6. Comparison with Prior Methods

Comparison with some related methods in recent literatures, aspects such as the principle, detection accuracy, detection accuracy, sampling rate, etc. are contrasted to evaluate the HCCNN method and show the properties, as summarized in Table 4.

In respect to principle of diagnosis, HCCNN uses the HFC sensors to collect high-frequency coupling signal. It does not set the threshold value in advance but is based on data supervised learning. It can also detect which load onsets an arcing fault and give a probability distribution. On the one hand, according to the relevant arc fault detection standards, it is not necessary to identify the load type, but it can help users to find the approximate position of the arc fault, which may be a research interest in the future. On the other hand, the load identification method is one of the major technical difficulties of non-intrusive composite load monitoring (NILM). The proposed method can be combined with NILM technology. Through the NILM, the power grid company can understand the actual energy consumption level of various loads of users, master the power consumption situation and behaviors of all kinds of users in real-time, guide users to reasonably arrange power consumption time, and provide energy-saving suggestions.

In the aspect of detection accuracy, the goal of each method is to identify the arc fault perfectly. The HCCNN method has higher precision (above 99%) than other methods and could be even higher by adjusting parameters perfectly. If a diagnosis scheme has an accuracy rate of more than 99%, it is generally believed in meeting the design requirements.

In the aspect of application range, the HCCNN method could be applied to switching, inductive, resistive, capacitive loads—more general than the methods in [19,20], which is not available to inductive loads application [19] or switched mode loads [20].

With respect to sampling rate, the HCCNN has a high sampling rate, and the highest sampling rate can be set to 16 MS/s because its hardware implementation is based on the FPGA target and high-speed ADC.

## 7. Conclusions

A study of series arc fault detection using the high-frequency coupling convolution neural network (HCCNN) method has been presented in this paper. The method is dedicated to series arc fault detection in a domestic network (220 V, 50 Hz), and detects arc fault in the following procedure: The gray images of various loads are obtained by using the designed high-frequency coupling (HFC) sensor that has the advantages of simple structure and wide frequency response range. A three-layer artificial neural network based on convolution neural network is designed to accurately identify series arc and load, as well as the load type.

Seven different domestic appliances, including a desktop computer, vacuum cleaner, induction cooker, fluorescent lamp, dimmer, heater, and an electric drill, generate an extensive generalized database for training and testing of the HCCNN method. The identification of arc fault can rely on the frequency information from 10 kHz to 500 kHz.

The experimental results confirm the HCCNN method’s effectiveness and good accuracy for series arc fault detection. If the load type is ignored, the detection accuracy reaches above 99%, and the accuracy of the electronic load test is also more than 99%. Besides, the detection method is currently implemented on an embedded system based around a Zynq-7020 FPGA microcontroller, and the time response is 11 ms for one sample.

## Figures and Tables

**Table 1:** The detailed parameters of the CNN layer and fully connected layer.

No.	Layer Type	Activation	No. of Kernel	Padding	Kernel Size	Stride	Output
1	Conv.	ReLU	8	2	5 × 5	1	8 × 100 × 100
2	Max-Pooling	-	-	-	2 × 2	2 × 2	8 × 50 × 50
3	Conv.	ReLU	16	2	5 × 5	1	16 × 50 × 50
4	Max-Pooling	-	-	-	2 × 2	2 × 2	16 × 25 × 25
5	Linear	-	1	-	16 × 25 × 25	-	16 × 25
6	Linear	Softmax	1	-	16 × 25	-	14

**Table 2:** Typical loads and the corresponding data set labels.

Appliance	Power	Model, Manufacturer	Data Set Label
Electric heater	1.2 kW	HZ-04, WAHIN	Normal: 0, Arc: 1
Vacuum cleaner	800 W	D1108, Bull	Normal: 2, Arc: 3
Computer	450 W	Lx-9, Haier	Normal: 4, Arc: 5
Dimmer	600 W	ZCY-08, Midea	Normal: 6, Arc: 7
Fluorescent lamp	40 W	40YT-MR, Philips	Normal: 8, Arc: 9
Induction cooker	1.8 kW	TW12-B2, Konka	Normal: 10, Arc: 11
Electric drill	1.2 kW	B5-JB02, Bosch	Normal: 12, Arc: 13

**Table 3:** Classification results of the HCCNN method.

Category (Normal)	0	2	4	6	8	10	12
Identification accuracy	99%	100%	100%	100%	100%	99%	100%
Category (Arcing)	1	3	5	7	9	11	13
Identification accuracy	99%	100%	98%	98%	94%	92%	98%
Prediction accuracy of each sample: 98.36%							
Prediction accuracy (ignore the load type): 99.20%							

**Table 4:** Comparison of the HTFNN with Prior Methods.

Method	Wang et al. [15]	Lezama et al. [20]	Tisserand et al. [19]	Yang et al. [27]	HCCNN Method
Principle	Fundamental frequency components and fully connected neural network.	The inter-period auto correlation coefficient of the line current.	The algebraic derivative method of the line current.	Using current transformer and convolution neural network.	Design of high frequency coupling sensor and convolution neural network.
Detection accuracy	Above 99.0% (3919 out of 3950 tests)	Not introduced in the paper.	Not introduced in the paper.	98.7% (987 out of 1000 tests).	99.2% (23 out of 1400 tests)
Sampling rate	20 kHz	5 kHz	1 MHz	1 MHz	1 MHz
Application range	Resistive, inductive, capacitive, and switching loads.	Resistive, capacitive, switching loads, but no inductive loads.	Resistive, reactive load, but no switching loads.	Resistive, inductive, capacitive, and switching loads.	Resistive, inductive, capacitive, and switching loads.
Device implementation assessment	The algorithm is implemented by STM32F407ZG and the time response of the method is 3 ms for one sample.	The algorithm is implemented by LCP1768 and the time response of the algorithm is 10 ms.	Declares that the implementation in FPGA has been implemented but few details about it.	Not introduced in the paper.	The method is implemented by Zynq-7020 and the time response of the method is 11 ms for one sample.
